# A report from a conference jointly organised by the European Academy of Cancer Sciences and the Pontifical Academy of Sciences, The Vatican, November 16–17, 2018†

**DOI:** 10.1002/1878-0261.12436

**Published:** 2019-02-27

**Authors:** European Academy of Cancer Sciences

## Abstract

Cancer is a massive challenge with a significant impact on society, healthcare systems, the economy and an increasing number of patients and their families. To help meet this societal challenge, the European Commission has recently proposed a mission‐oriented approach to cancer in Horizon Europe, and about 60 participants met at the Vatican to discuss a mission‐oriented approach to cancer in Europe, as documented in this report. Painting: Rowe, Ernest Arthur. *Courtyard of the Casina Pio IV* (ca. 1911–1913).

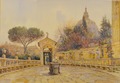

1

Cancer is a massive challenge with a significant impact on society, healthcare systems and the economy, as well as an increasing number of patients and their families. At present, there are 4.23 million new cancer patients in Europe annually, 1.94 million deaths, and an increasing number of patients living with a cancer diagnosis. To help meet this human and societal challenge, the European Commission has recently proposed a mission‐oriented approach to cancer in Horizon Europe, since fighting cancer using a mission approach is likely to change the lives of many families across Europe and beyond.

About 60 participants met at the Vatican (https://www.europeancanceracademy.eu/user_uploads/files/EACS%20PAS%20Vatican%20conference%20Nov%202018.pdf) to discuss a mission‐oriented approach to cancer in Europe, stating that by combining innovative prevention and treatment strategies in a sustainable state‐of‐the‐art virtual European cancer centre/infrastructure**,** it will be possible by 2030 to achieve long‐term survival of 3 out of 4 cancer patients in countries with well‐developed healthcare systems. Furthermore, concerted action will pave the way for better handling of economic and social inequalities in countries with less‐developed systems (Celis and Pavalkis, 2017).

Marcelo Sánchez Sorondo, Bishop‐Chancellor of the Pontifical Academy of Science, and Alexander Eggermont, President of the European Academy of Cancer Sciences, formally opened the conference by welcoming the participants to the Vatican (Fig. 1). Msgr. Sánchez Sorondo said that the increase in cancer, which now also affects children and young people, shows that the enormous dispersion of research and funds has not contributed to solve the problem of cancer, in contrast to other diseases. This situation obliges us to rethink the strategy to combat cancer, avoiding this dispersion and unifying research efforts to promote prevention and identify causes of disease, according to the aim of this workshop.

**Figure 1:**
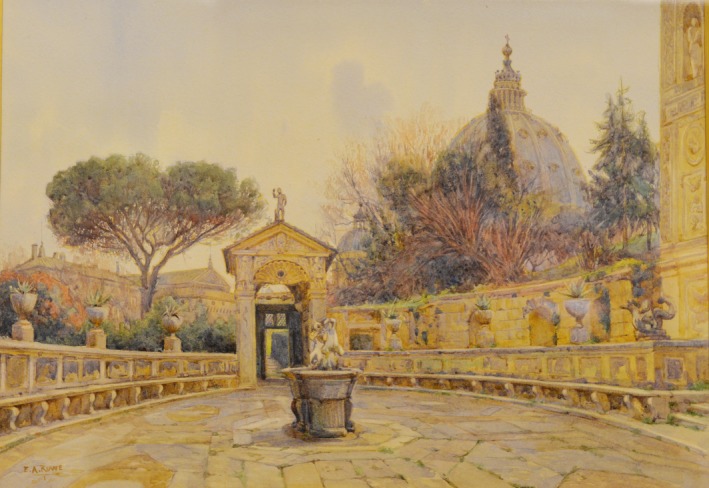
Courtyard of the Casina Pio IV. (ca. 1911–1913). Artist: Rowe, Ernest Arthur

## 2 Opening session: ‘Towards increasing the social impact of cancer research’

The session began with a presentation by Julio E. Celis who provided a brief account of the steps leading to a potential cancer mission in Horizon Europe. He told the audience that the mission was rooted in the efforts taken by both the European cancer community and the European Commission during the last 16 years to address the fragmentation and lack of coordination of European cancer research. These activities led to the creation of a pan‐European platform/infrastructure for translational cancer research – including Cancer Core Europe and Cancer Prevention Europe (Eggermont *et al*., 2014; Forman *et al*., 2018) – that will be composed of interlinked cancer research centres with shared infrastructures and collaborative projects to facilitate rapid advances in knowledge and their translation into better cancer treatment, care and prevention. He also emphasised the role of Comprehensive Cancer Centres (CCCs) being entities that are patient‐focused and connect research and education with care and prevention, thereby linking research with the healthcare system. Concluding, Julio E. Celis pointed out that the Commission recently put forward to the Competitiveness Council a cancer mission centred on paediatric oncology and suggested that this outcome should be discussed at the conference since the scientific, social and economic impact of a restrictive mission will be limited.

In a video recording, Commissioner Carlos Moedas told the audience that ‘Europe has always been a global centre for innovation, so it makes sense that we are at the forefront of innovative approaches to address cancer. Cancer has evolved, and we need new tactics to match it. In Horizon Europe we want to introduce mission‐oriented research to set an inspirational goal to direct our research efforts towards a concrete objective and to get the public involved’. He addressed the importance of the missions, and in particular, he stated: ‘missions should inspire people’, and he was sure that ‘a mission on cancer would appeal to millions of European citizens because it will relate to all of us, especially, if we talk about children’. Also, he told the participants that missions should deliver results and act at the European level. An EU‐level mission in cancer would make sense, and if the Member States and the European Parliament agree with the Commission proposal, the support of the whole cancer research community will be needed to change the lives of cancer patients. He also promised to fund cancer research further beyond missions.

Commissioner Vytenis Andriukaitis welcomed the initiative of the conference organisers to gather some of the world's leading oncological researchers, physicians and policymakers with the aim of highlighting the social impact of cancer research. We know the challenge of fostering research and developing new medicines while maintaining the balance between access and affordability for patients, business viability for companies, and the sustainability of health systems. We must keep working towards achieving these goals. The new technologies at our disposal usher in the promise that we can make tangible improvements for cancer patients and survivors, particularly if we pool our resources across sectors and national borders.

The European Commission has been supporting such collaborative efforts with, for example, the recent Communication on Digital Health and Care and the Health Technology Assessment proposal. If we bring together and share our limited data sets and research results, we can expand their potential benefit to many more patients. In all of this work, we must keep the patient at the centre. After all, positively influencing patients and communities can boost the social impact of innovative cancer research.

In a video recording, the President of the European Parliament (EP), Antonio Tajani, stressed that cancer is our primary health challenge and that we must tackle it together with the EU, the scientific community, patient associations and citizens as partners. The fight against cancer has always been a top priority of the EU, spending €200 billion on research since 1984 via the Framework Programmes to bring together scientists from Europe and around the world, the latter being central to the concept of science diplomacy to be discussed later at the conference.

He also reminded the audience that the number of new cancer cases in the EU would significantly increase annually; thus, we could not leave European citizens alone. He reiterated that research was still at the core of Europe's policy and that the EP has pressed for a 3% of GDP research investment target across the EU by 2020.

Concluding, President Tajani reminded us that Pope Francis taught us four keywords: prevent, repair, cure and prepare for the future, telling us that these principles should embrace our mission. The research of today will build the world of tomorrow and investing in research is investing in our future! To achieve this goal, we need your support.

Minister Manuel Heitor stressed the importance of a mission in cancer and pointed out that a mission‐oriented approach must involve a process guaranteeing the involvement of the research community and should impact Europe at large. The Vatican conference clearly shows the need to adequately include cancer prevention in order to mitigate the burden of cancer for patients and society. Also, policy instruments at EU and national levels must promote further interactions between cancer centres, creating networks of CCCs throughout Europe characterised by research excellence.

## 3 Session: ‘Scientific strategies towards a mission‐oriented approach to cancer’

Ulrik Ringborg gave an articulated overview of the complexity of the cancer research continuum (see also review by U. Ringborg in this issue; Ringborg, 2019). He believes that concerted action is urgently needed to link prevention (to decrease incidence) with early detection and treatment to increase the cure rate; furthermore, personalised/precision cancer medicine in which treatment is tailored to the biology of a tumour is required to increase the cure rate, prolong survival and improve health‐related quality of life. In therapeutics, translational cancer research is by definition focused on patients’ problems and is defined as a consistent research continuum from basic/preclinical research to early clinical research, late clinical research and following adoption by the health care to outcomes research. This research continuum still has a number of gaps that must be bridged to reach consistency from basic/preclinical research to outcomes research. For prevention, there is a similar research continuum with its own specific lacunas. Achieving a coherent translational research path for both therapeutics and prevention is mandatory to integrate multidisciplinary cancer research and intensify innovation.

Alexander Eggermont presented Cancer Core Europe, a European legal entity consisting of seven leading cancer centres – most of them Comprehensive Cancer Centres (CCCs) – with a single portal system to engage in various research projects with partners (Calvo *et al*., 2018). Cancer Core Europe was established as an auto‐financed bottom‐up network to create a sustainable, high‐level, shared research infrastructure platform facilitating research collaborations and setting up taskforces (data sharing, clinical trials, genomics, immunotherapy, imaging, education and training, and legal and ethical issues), with a carefully planned expansion agenda to eventually engage with additional European countries and with other European networks in variable geometries. Cancer Core Europe is committed to be part of a network of networks brought together by a future mission. Translational cancer research covers the cancer research continuum from basic/preclinical to early clinical, late clinical and outcomes research. Basic–preclinical research is the ‘engine’ for early clinical research in personalised/precision cancer medicine, bridging the early translational research gap, and is currently the primary focus of Cancer Core Europe as exemplified by the launching of the Basket of Baskets (BoB) trial, Europe's largest precision cancer medicine trial.

Christopher P. Wild presented the Cancer Prevention Europe (CPE) consortium (Forman *et al*., 2018). A striking rise in the cancer burden in Europe is projected for the coming decades, driven by an ageing population and an increased exposure to risk factors. In parallel, costs are spiralling for the treatment and care of patients and long‐term survivors. The resulting health and economic costs pose a serious threat to the sustainability of health services. Indeed, no country can afford to treat its way out of the cancer problem. Instead, a balanced and integrated approach is required encompassing prevention, early detection and treatment. Furthermore, all cancer control measures should reduce rather than exacerbate inequalities among European citizens, so that no one is left behind. However, while cancer prevention is essential, it is frequently underemphasised and under‐resourced in comparison with the development and implementation of new treatments. The creation of the CPE consortium provides the requisite interdisciplinary expertise needed from across Europe to produce a strategic vision for cancer prevention research, implementation and advocacy.

Presently, Cancer Core Europe and CPE are in the process of developing complementary therapeutics and prevention strategies to address in partnership the growing cancer problem. By providing innovative approaches for cancer research, links to the healthcare systems, development of quality‐assured multidisciplinary cancer care, and the assessment of long‐term outcomes, these initiatives provide a proof of principle in bringing together the required expertise to address the major challenges in addressing the cancer burden in Europe.

Anton Berns, chair of the standing committee of the European Academy of Cancer Sciences (EACS) that oversees the Designation of Excellence (DoE) programme (Ringborg *et al*., 2018), emphasised the importance of incentivising cancer research institutions to boost high‐quality, innovative research efforts. This requires an overhaul of how we organise cancer research. CCCs are best positioned to catalyse these developments, provided these centres have sufficient critical mass and put quality and innovation as their top priority. Programmes of the Organisation of European Cancer Institutes (OECI), German Cancer Aid and the DoE of the EACS must play an important supportive role to improve translational cancer research and encourage collaboration between CCCs. However, to effectively execute innovative, data‐rich clinical trials, these networks of CCCs will also have to establish closely collaborating clusters that develop and disseminate unique expertise. In this way, a variety of distinct and complementary geometries can arise each with sufficient critical mass to make substantial contributions, and the mission can provide the necessary financial support required for the CCC networks to succeed and for OECI and EACS to be sustainable pan‐European organisations that have a strong focus on accreditation. By promoting a number of clusters rather than establishing an overarching virtual institute with many members, a mission would support a pan‐European network of networks and organisations that remains flexible and manageable. Nevertheless, such clusters should as much as possible align their standard procedures. This will facilitate the execution of data‐rich innovative clinical trials and permit these clusters to serve as valuable contact points for pharma and larger scale collaborations with other clusters. Cancer Core Europe and Cancer Prevention Europe represent valuable but not exclusive examples of such initiatives, and the mission could provide an opportunity for these developments to be sustainable.

Funding mechanisms for building of a plethora of geometries that properly cover the cancer research continuum at a high‐quality level are not available. Also, some areas of the cancer research continuum are under‐resourced (e.g. prevention, early detection and outcomes research), and substantial strides should be made for implementation (measures in the area of prevention that can be taken now!). A mission would be an opportunity to fill these gaps.

Harald zur Hausen discussed infections and cancer biology with the aim of developing new strategies for primary prevention based on discoveries of causes of cancer. He was awarded the Nobel Prize for his discovery of human papillomavirus as a causative agent for cervical cancer, the basis for the vaccination programme to prevent this disease. As an example of research linking epidemiological demonstration of risk factors to the identification of the causes of cancer in biological terms, Harald zur Hausen presented ongoing work exploring the background of red meat and dairy products as risk factors for several tumour diseases, among them colorectal and breast cancer. Small circular single‐stranded DNA genomes have been isolated from Aurochs‐derived Eurasian dairy cattle sera and milk products and have also been observed in tumours of patients with colorectal and breast cancer. He proposed that infection early in life with ‘Bovine Milk and Meat Factors’ may result in chronic infection with chronic inflammation as an indirect cause of cancer. The presentation of his ongoing work illustrates the complexity of linking risk factors identified by epidemiological research to biologically defined causes of cancer, and he stressed the importance of expanding research on causes of cancer as a basis for primary prevention strategies.

Jeffrey D. Sachs from Columbia University, NY, discussed the critical issue of achieving financial support for innovative research. With examples from the United States, he illustrated different economic outcomes depending on private or public funding. The latter has a special bearing on research intended to deliver new anticancer agents, a research area where the pharmaceutical industry currently invests more than in other medical areas. In the discussion, it was stated that incentives for patient‐oriented research or research for prevention should not be based on economic interests, but on patients’ needs and the creation of cost‐effective prevention and health care. With these ambitions, public funding should have priority. Jeffrey Sachs also pointed out that low financial support at the EU level is a problem when international collaborations are mandatory for research to create innovation. He also pointed out that the current patent system is ‘broken’ as far as it regards the development of new anticancer medicines and that it does not serve society as it should.

## 4 The presentations were followed by a general discussion

### 4.1 Scientific strategies towards a mission‐oriented approach to cancer

Anton Berns moderated the discussions, and the following questions were put forward to the audience: Why is a pan‐EU broad cancer mission important? Why is a coherent cancer research continuum important? Why is reaching or retaining critical mass an increasing problem? Is the support for the different components of the research continuum properly balanced? How can we assure that all member states will benefit?

### 4.2 Social impact of cancer research

Ulrik Ringborg moderated the discussion and raised the following questions: Which are the most important unmet needs of patients and which are the main challenges for healthcare systems? How can we best secure the regular updating of multidisciplinary guidelines and harmonise protocols for assessment of the quality of care, clinical effectiveness, cost‐effectiveness and quality‐of‐life parameters? Can CCCs play a special role in this? How to reach all patients? How can we cope with increasing cancer survivorship? How to best communicate to society the benefit of science, empower patients, and invigorate social innovation?

### 4.3 Science diplomacy

Cecilia Björner and Jan Tombinski moderated the discussion raising the following questions: What is science diplomacy and what are the challenges? How can we use science diplomacy to orchestrate a pan‐EU cancer mission? Research is a competence of the EU, but health is not. How can we align policies and priorities? The Comprehensive Cancer Centre is a unique entity integrating research with cancer care and prevention. How can we achieve at least one CCC per country and one per 5–10 million inhabitants in large countries? There are inequalities both within a country and between countries regarding cancer prevention, cancer care, research and education. What is required for a unified vision and strategy to decrease the inequalities? How do we engage other continents to make an EU cancer mission ‘open to the world’?

Both the presentations and the discussions indicated that the cancer community is united, well motivated and prepared for a mission‐based approach on cancer.

The following main conclusions were reached:

- The current negative trend of an increasing cancer burden can only be reversed by giving priority to excellence in research, the engine that fuels innovation in both therapeutic and prevention activities.
- CCCs, entities that link research and education with prevention and treatment, are considered essential to catalyse this process as they connect research with the healthcare systems and integrate every step in the cancer research–care continuum from basic/preclinical, to early clinical, late clinical and outcomes research.
- Accreditation methodologies for quality assurance, both of multidisciplinary care and of research, are already validated and should be further supported to be more broadly implemented and continually improved. These accreditation schemas are fundamental for spreading and objectively evaluating innovations and provide the basis for health economics assessment.
- Prevention complements advances in treatment and care. It is estimated that around 40% of cancers could be prevented, if current knowledge about cancer risk factors was better translated into primary prevention strategies. Cancer screening and other types of early detection can further reduce the incidence of cancer and improve survival through more effective treatment of early‐stage disease.
- Cancer is characterised by inequalities in outcomes between countries and between population groups within countries, reflecting differences in access to prevention, early detection, treatment and care. It is important that all cancer control initiatives are designed to ‘leave no one behind’.
- Closely collaborating consortia of cancer research centres and research networks are required to achieve the critical mass of resources, expertise and patients required for innovation. All EU countries must have mechanisms to participate, associate with and benefit from these networks.
- The balance between public and private funding of clinical and translational research should shift more towards public funding, in particular of clinical trials across Europe. The support for prevention research from public funding needs to be substantially increased.
- A mission on cancer is linked to important policy questions that must be expressed in political terms. The role of diplomacy to achieve an effective ‘cancer mission’ both within the EU and in the context of ‘open to the world’ was discussed, the latter of which received very positive support from representatives of other continents.
- The European Commission recently suggested restricting the cancer mission to paediatric oncology. The conference, however, concluded that as cancer is predominantly a disease of ageing, focusing on the 0.6% of all cancer patients in the 0‐ to 19‐year age group will severely compromise the scientific, social and economic impact of a mission. Moreover, a mission limited to paediatric oncology would not address research in prevention and early detection, which are of fundamental importance to make progress against cancer overall. Paediatric oncology should, however, be an essential component of a mission covering all cancers.
- The conference at the Vatican, jointly organised by the European Academy of Cancer Sciences and the Pontifical Academy of Science, conveys an important symbolic message linked to ethics, social justice and human rights by emphasising our obligation as a cancer research community to reduce the suffering from cancer by giving priority to research in cancer prevention and effective treatments, this to the benefit of all citizens in Europe.

## Author contributions

All authors contributed to the writing of this report.

## Conflict of interest

The authors declare no conflict of interest.
